# Cardiology trainees' attitudes towards clinical supervision: a scale development study

**DOI:** 10.5116/ijme.604a.4964

**Published:** 2021-03-26

**Authors:** Iswandy Janetputra Turu' Allo, Anwar Santoso, Ardi Findyartini

**Affiliations:** 1 Medical Education Centre, School of Medicine, The University of Nottingham, Nottingham, UK; 2 Department of Cardiology-Vascular Medicine, Faculty of Medicine, Universitas Indonesia/National Cardiovascular Centre-Harapan Kita Hospital, Jakarta, Indonesia; 3 Department of Medical Education, Faculty of Medicine, Universitas Indonesia, Jakarta, Indonesia

**Keywords:** Cardiology training, clinical supervision, scale development

## Abstract

**Objectives:**

This study aims to explore the construct validity, dimensionality, and internal consistency of a new attitude scale for measuring cardiology trainees' attitudes towards clinical supervision.

**Methods:**

A multi-centred, cross-sectional study involving 388 Indonesian cardiology trainees from eight universities was conducted using convenience sampling. Twenty-nine items have been generated based on an extensive literature review and conceptual framework of effective clinical supervision. Ten clinical experts reviewed the items to ensure the Cardiology Clinical Supervision Scale (CCSS) adequately represents the construct under study. An exploratory factor analysis using principal axis factoring (PAF) with oblique rotation was run to identify the internal structure of the scale. Items with factor loading <0.50 were deleted. In addition, inter-item correlations and items' communalities were analysed. Each subscale's internal consistency was assessed using Cronbach's alpha score.

**Results:**

The content validity index provided evidence for CCSS' validity (G-coefficient=0.71). Scrutinising the experts' comments, we finalised the scale to include 27 items. Further, four items were deleted due to low inter-item correlation and communality. PAF analysis resulted in a two-factor model comprising the "Supervisory Interaction and Facilitation" factor (n=10 items) and the "Role Modelling" factor (n=9 items); four items were deleted due to low factor loading. The Cronbach's alpha score for SIF and RM factors were 0.93 and 0.89, respectively.

**Conclusions:**

The study's results support the validity, internal structure, and internal consistency of the new clinical supervision scale for cardiology training. Further studies are required to investigate other validity and reliability evidence for CCSS, including its cross-cultural validity.

## Introduction

Clinical supervision is an integral part of medical training which can improve patients' safety and enhance the educational outcome of the trainees.^1^ From the educational perspective, it serves as facilitative learning, based on trainees-supervisors relationship^2^^,^^3^ and provides progressive independence^4^ and development opportunities, while maintaining standards of practice^5^ and ensuring safe environments for both patients and trainees.^2^^,^^6^ More importantly, during clinical supervision, trainees may observe and model their clinical supervisors' behaviours for their roles as future supervisors.^7^

Given the importance of clinical supervision, some specialities such as internal medicine,^8^^-^^11^ geriatric medicine,^12^ psychiatry,^13^ emergency medicine,^14^^,^^15^ surgery,^16^^,^^17^ anaesthesiology,^18^^,^^19^ and general practice^20^ have developed scales for measuring clinical supervision in their postgraduate training. Although studies have provided validity and reliability evidence for these scales using different psychometric approaches, most of them are lacking in one or more indicators (i.e., items), which are necessary for effective clinical supervision.

Although the principles of effective clinical supervision may be similar across specialities,^2^ it is a question of validity whether the scales developed for other fields can measure what is intended to be measured for cardiology trainees. Beckman and colleagues^21^ in their study have found that a scale developed for internal medicine might not be valid for cardiology trainees in the same institution. Differences in the educational environment, the nature of the specialities, and the types of patients encountered daily by cardiologists (and their trainees) and internal medicine specialists may be the reason for the invalidity of this scale when used by cardiology trainees.^21^ This indicates that cardiologists and medical educators need to develop items to reflect cardiology training more accurately.

Moreover, as for other specialities, for cardiology, the importance of quality clinical supervision and its evaluation have been recognised by postgraduate cardiology training bodies.^22^ The use of psychometrically sound items to represent the relationship between items and the construct being measured (i.e., clinical supervision) is, therefore, required for evaluating clinical supervision practice in cardiology training. However, to the best of our knowledge, there is no clinical supervision scale to measure cardiology trainees' attitudes towards clinical supervision.

Therefore, we find that it is essential to measure clinical supervision from the perspectives of cardiology trainees using a valid and reliable instrument. Intended to develop such a scale, this study was informed by a key conceptual framework of effective clinical supervision explained in the literature as including: (i) a supervisor's dedication, time and availability,^23^^,^^24^ (ii) clarity and specificity of the task and objectives at hand,^25^ (iii) trainees' autonomy changing throughout training,^26^ (iv) a quality supervisory relationship,^2^^,^^25^ (v) a supervisor's positive attitude and professional capability,^27^ (vi) reflective practice,^28^ and (vii) accurate, balanced, and timely feedback.^29^^,^^30^

This study aims to develop a scale to yield valid and reliable scores for measuring cardiology trainees' attitudes towards clinical supervision. Such a scale will improve the current practice of clinical supervision in cardiology training and provide a means for measuring and monitoring the quality of cardiology training.

## Methods

### Study setting

Indonesia has 13 state-owned universities for postgraduate cardiology training. A four-year training program is conducted at state-owned hospitals affiliated with each university. Each trainee or group of trainees has a principal clinical supervisor in each sub-division. However, trainees are allowed to practice under the supervision of other consultants when necessary. In Indonesia, the standard ratio between supervisor and trainees is 1:5, i.e., one supervisor for five trainees.^31^

### Study design and participants

A multi-centre, cross-sectional study was conducted to examine the validity and reliability of a newly developed Cardiology Clinical Supervision Scale (CCSS) to measure cardiology trainees' attitudes towards clinical supervision. In this study, the sample consisted of cardiology trainees from eight out of thirteen universities in Indonesia, where postgraduate cardiology training is conducted. For data confidentiality purposes, the universities have been anonymised and referred to as university A to H. Using a convenience sampling approach, 388 responses were collected.  Table 1 shows the frequency distribution of cardiology trainees by demographics. Ethical approval for this study was obtained from the research ethics committee of the University of Nottingham, UK and Universitas Padjadjaran, Indonesia.

**Table 1:** Demographic profile of the study participants (N=388)

Variable	Frequency (n)	Percentage (%)
Gender		
Female	159	40.98
Male	229	59.02
Total	388	100
University		
A	46	11.86
B	64	16.49
C	67	17.27
D	32	8.25
E	30	7.73
F	69	17.78
G	28	7.22
H	52	13.40
Total	388	100
Year of Training		
1^st^ Year	59	15.21
2^nd^ Year	95	24.48
3^rd^ Year	95	24.48
4^th^ Year	73	18.81
5^th^ Year	52	13.40
>5^th^ Year	14	3.61
Total	388	100

### Generating items of the CCSS scale

A total of 29 preliminary items were generated based on an extensive examination of the literature and the conceptual framework of effective clinical supervision given above (e.g., the item on reflective practice was supported by Launer^28^). Of those 29 items, three items were negatively worded (e.g., "My clinical supervisor treats the trainees unequally"). Negative statements were included to prevent acquiescent bias or extreme score bias.^32^ Response options were set using a five-level Likert Scale of 1, 2, 3, 4 or 5, corresponding to 'strongly disagree', 'disagree', 'neutral', 'agree' and 'strongly agree', respectively, to measure the trainees' attitudes towards each item.

### Content validity

To evaluate the scale's content validity, ten experts reviewed the relevance of each item within the scale using a five-point Likert scale. Also, they were invited to provide comments on how to improve the items and the scale as a whole.  Each item's clarity and consistency within the conceptual frameworks were also crucial to review. Based on the experts' interpretation, the content validity index (CVI), which shows the extent of the experts' agreement, was calculated.^33^ The alpha coefficient^34^ was calculated as the index of the content validity when more than two judges rated the scale.^35^ It is worth noting that the alpha coefficient is identical to a single-facet generalisability (G) coefficient (Judges × Items). Within a G-study, using a single facet design, researchers not only obtain the alpha coefficient but also explore the variance components for each facet (i.e., experts) and each item and the interaction between experts and items.^36^ Furthermore, all experts' comments were reviewed and addressed to improve these items.

### Data collection method

The Collegium of Cardiology and Vascular Medicine in Indonesia advised the cardiology departments to allow trainees to participate in the study. Program Directors in all centres agreed to participate in the study and assigned one trainee as a contact person. Next, an email invitation was sent to all trainees along with a unique JISC Online Survey (formerly Bristol Online Survey) link (bound to each email address), which allowed them to use the link only once. To increase the response rate, the contact persons were asked to encourage trainees to join the study. Moreover, four reminder emails were sent to the trainees. After the conclusion of data collection, the trainees' emails were deleted, and they were converted to specific codes to ensure participants' anonymity.

### Factor analysis

Factor analysis (FA) is a powerful statistical technique for examining the association between observed and latent variables based on items' correlation.^37^ Items that are correlated strongly are joined, forming a factor or dimension. Using FA, we pinpoint, isolate and estimate these factors. FA is usually split into two major parts: (i) exploratory factor analysis (EFA), which is used when the relationship between the observed and latent variables and the number of factors is not clear, and (ii) confirmatory factor analysis (CFA) which is used when the researcher understands a scale's factor structure based on a theory or previous study, including prior EFA analysis.^38^

When using EFA, we need to determine whether to use Principal Axis Factoring (PAF) analysis, also known as Principal Factor Analysis or Principal Component Analysis (PCA). Although the theoretical principles of PAF and PCA differ, they produce quite similar results.^39^^,^^40^ EFA with PAF was used in this study in order to identify the latent constructs behind the items, which is aligned with the objective of our study. It should be noted that the PCA approach is chosen if researchers wish to reduce the numbers of items (i.e., observed variables).^40^

### Data analysis

Given the purpose of this study is to identify the factor model of the CCSS scale in measuring clinical supervision, EFA using PAF with promax (i.e., oblique) rotation was conducted. To achieve these factors, several steps were performed as follows: first, the assumptions of the FA approach were assessed before the data were analysed using Kaiser-Meyer–Olkin (KMO) statistics and Bartlett's Test of Sphericity. Next, the correlation of items, extracting factors, oblique rotation (i.e., promax) and interpretation of factors and the reduction of items using factor loadings were applied. Other psychometric methods used were inter-item correlation and corrected item-total correlation. If the correlation between the two items was higher than 0.30, they were retained. Further, items that showed a low communality (<0.40), or which had an unclear meaning relative to other items were identified to be removed. The eigenvalue was used to drive the factors. Factors with an eigenvalue greater than one were compared with the results of the scree plot to obtain a better picture of the factors.

To maximise factor loading, an oblique rotation method was used. This is because there is an opinion amongst researchers that factors are likely to correlate with each other.^41^ However, we also did an orthogonal (i.e., varimax) rotation to compare the factors solution yielded by both rotations. Each item's factor loading was assessed to identify the latent construct. A factor loading of 0.50 was chosen as the threshold, being a score falling between 0.45 (good) and 0.55 (very good).^42^ In terms of the cross-loaded items with a factor loading difference between two or more factors ≤0.20,^43^, the items' conceptual meanings were examined to decide the most suitable factor with which to place them.^44^ However, if we were unable to determine with which factor to put the item, the particular item would be discarded.^43^ Cronbach's alpha was calculated for subscales to assess the reliability of the scale scores. An alpha of 0.70 or higher showed that the reliability of the scale scores was satisfactory.^45^

## Results

### Initial items and content validity of the CCSS

As presented, 29 items were initially developed based on the conceptual framework of clinical supervision in this study, including three negatively worded items. The G-coefficient of the single-facet G study from ten experts showed a satisfactory agreement between experts (G-coefficient=0.71). Based on the experts' comments, several amendments were made to items. Two items were merged into one, two items were deleted, and one item was added based on an expert's suggestion which was well suited to the study's conceptual framework. At the end of the content validity analysis, CCSS had 27 items, including two negative statements.

### Exploratory factor analysis

Data adequacy analysis indicated an adequate amount of data for FA (KMO analysis 0.96 and Bartlett's Test of Sphericity χ^2^(351, N=388)=6071.22, p=0.00)). An inter-item correlation matrix showed that item 9 and item 15 had a correlation coefficient of less than 0.30. The corrected item-total correlation ranged from 0.33 (item 9) to 0.78 (item 20). Therefore, items 9 and 15 were removed from the analysis. Item communalities were scrutinised to detect underperforming items. Items 3 and 26 showed a low communality (<0.40). Consequently, items 3,9,15 and 26 were deleted from further analysis.

Putative factor extraction using PAF has yielded two factors with an eigenvalue greater than 1. The scree plot (the factors are plotted against the eigenvalue) also supported the thesis that these two meaningful factors explain most of the variance, and a third factor would only explain an insignificant amount of variance, and hence was not retained.

A promax (i.e., oblique) rotation based on a two-factor solution showed that most of the items loaded >0.50 into one factor. Exceptions were item 24, item 21, item 16, and item 8, which loaded <0.50 to both factor 1 and factor 2. Therefore, items 24, 21, 16, and 8 were deleted from the scale. The CCSS consisted of 19 items (factor 1: 10 items and factor 2: 9 items) after completion of the factor analysis. Table 2 shows the two factors with their percentage of explained variance and each item's descriptive statistics. As we can see from Table 2, the mean item scores ranged from 3.49 to 4.33, and the item communalities range from 0.42 to 0.70. This table also shows that two factors explained 57.35% (51.05%+6.30%) of the variance in the data set. Therefore, we retained 19 items based on the FA of the original 27 items. After scrutinising each item in both factors in the light of the conceptual framework, we labelled factor 1 as "Supervisory Interaction and Facilitation" (SIF), consisting of 10 items, and factor 2 as "Role Modelling" (RM), consisting of 9 items.

### Scale's reliability

The CCSS consisted of 19 items with two subscales. The SIF subscale consisted of 10 items (Alpha=0.93), and the RM subscale consisted of 9 items (Alpha=0.89). Table 3 presents each factor's reliability score and descriptive statistics.

## Discussions

### Summary of findings

This study aims to develop a new scale for measuring cardiology trainees' attitudes towards clinical supervision and evaluate its validity and reliability. A satisfactory single-facet generalisability test (G-coefficient=0.71) conducted on 29 initial items proves that the items in CCSS measure clinical supervision. Furthermore, PAF analysis on 27 items (after content validity evaluation) yielded a hypothetical model consisting of 19 items separated into two subscales; (i) Supervisory Interaction and Facilitation (SIF) (10 items) and (ii) Role Modelling (RM) (9 items). Both factors had good Cronbach's alpha scores, 0.93 for SIF and 0.89 for RM.

### Content validity and internal structure of the CCSS scale

Content validity evaluation showed that the initial CCSS scale (consisting of 29 items) measures the tenets of clinical supervision and therefore was suitable for measuring what it was intended to measure. The construct validity evaluation yielded a two-factor model, which explained 57.35% of the total variance. The conceptualisation of each item loaded to each factor led us to label the first factor as Supervisory Interaction and Facilitation (SIF) (n=10 items) and the second factor as Role Modelling (RM) (n=9 items).  The label SIF was based on higher loading items in the first factor (i.e., items 25, 19, 4, and 18). Items 19 and 18 showed "supervisor-trainee interaction", whereas items 25 and 4 showed "supervisory facilitation" aspects. We labelled the RM factor based on the meaning shared by all items comprising it. Several of RM's items show the supervisor's role of modelling as a physician (e.g., items 5, 2, and 14), and the others (e.g., items 10, 11, and 13) indicate the supervisor's role of modelling as a supervisor. Both are role modelling tasks in clinical supervision as a supervisor needs to be an excellent example for trainees in their process of becoming physicians and future clinical supervisors.^7^

To achieve the best solution, both oblique (i.e., promax) and orthogonal (i.e., varimax) rotation were conducted. The two types of rotation produced similar results. However, varimax rotation yielded more cross-loaded items. Therefore, to achieve a simpler solution with better factor loading, promax rotation was used and reported in this study. The structure of the CCSS scale is, arguably, simpler to interpret and easier to utilise in the clinical supervision process by contrast with what has been developed in other scales used in other specialities, such as internal medicine. In the Wisconsin Inventory of Clinical Teaching (WICT),^8^ the supervisor's function as a role model has been divided into several sub-dimensions (e.g., "the attending doctor as a clinical role model" and "the attending doctor as a clinical supervisor"). However, in CCSS, these two dimensions have been blended into one factor (i.e., RM), based on factor analysis, which was not conducted in the development of WICT.^8^ Moreover, although it includes different items, the clinical teaching assessment instrument developed by Beckman and Mandekar^11^ might have a meaning similar to CCSS' factors. In their study, factor analysis produced a three-factor model (i.e., interpersonal domain, clinical teaching domain, and efficiency domain) when conducted on general internal medicine trainees. However, when it was tested on cardiology trainees, the interpersonal and clinical teaching factors were collapsed into one factor.^21^ In our study, the blended factor in their study seems to have a meaning similar to the SIF factor in CCSS. To what degree cardiology trainees can (and/or cannot) distinguish interpersonal interactions in supervision and in clinical supervision facilitation might be studied further.

### Scale's reliability

In terms of CCSS' reliability, both factors had good internal consistency, 0.93 for SIF and 0.89 for RM. Besides, all corrected item-total correlations were above 0.30, and the correlation between the two items were not less than 0.30, indicating that the items yielded are part of the scale. Cronbach's alpha was higher than 0.90 for factor 1 (SIF) and may imply a redundancy between items (i.e., testing the same variable but in a distinctive appearance).^45^

**Table 2:** Principal Axis Factoring of CCSS Scale using promax (oblique) rotation with communalities of each item (N=388)

Item^*^	Statement	Factor 1^†^	Factor 2^†^	h^2^	Mean	SD
25	My clinical supervisor is open to feedback about his/her supervision	0.99		0.62	3.49	0.85
19	My clinical supervisor understands my difficulties in training and provides helpful advice and support	0.91		0.70	3.66	0.78
4	My clinical supervisor evaluates my knowledge and skills and provides non-threatening, constructive, and timely feedback	0.70		0.59	3.73	0.81
18	My clinical supervisor treats me with respect	0.69		0.58	3.93	0.69
1	My clinical supervisor recognises my learning needs during supervision	0.67		0.52	3.79	0.77
12	My clinical supervisor develops a safe and confidential environment for my supervision	0.67		0.54	3.73	0.75
20	My clinical supervisor is enthusiastic, engaging, and committed to his/her role as a clinical supervisor	0.65		0.66	3.81	0.72
27	My clinical supervisor plans scheduled supervision sessions	0.62		0.42	3.49	0.88
22	My clinical supervisor assigns me to suitable patients according to my current competency and ability	0.53		0.56	3.81	0.74
7	My clinical supervisor encourages me to reflect on my clinical experiences	0.52		0.56	3.90	0.75
5	My clinical supervisor is up to date with the latest developments in cardiovascular medicine		0.80	0.45	4.33	0.68
2	My clinical supervisor follows good medical practice (e.g., putting patient safety first, shows professional behaviours toward patient and colleague)		0.73	0.50	4.21	0.64
10	My clinical supervisor challenges my clinical reasoning and problem-solving skills		0.70	0.44	3.98	0.73
6	My clinical supervisor shares his/her knowledge and experience with me		0.69	0.49	4.11	0.68
11	My clinical supervisor discusses complicated cases and patients' management plans with me		0.67	0.54	3.96	0.70
14	My clinical supervisor encourages me to collaborate with other health professionals in our team (e.g., other consultants, nurses, or pharmacists)		0.64	0.46	3.97	0.68
23	My clinical supervisor allows me to perform clinical procedures (invasive/non-invasive) according to my current competency and ability		0.64	0.42	4.08	0.62
13	My clinical supervisor acknowledges and answers my questions professionally		0.56	0.54	3.92	0.65
17	My clinical supervisor appreciates and values different cultures		0.55	0.49	3.93	0.63
Variance (%)		51.05	6.30			

^*^Total final items are 19 items; items number are 1-27 (item no 9 and 15 were deleted due to inter-item correlation <0.30; item 3 and 26 were deleted due to communalities <0.40; and item 8, 16, 21, and 24 were deleted due to factor loading <0.50). Items are sorted from item with the highest factor loading to the lowest in each factor.

^†^Factor labels are as follows: Factor I, Supervisory Interaction and Facilitation (SIF) (n=10 items) and Factor II, Role Modelling (RM) (n=9 items).

h2: item communalities; SD: standard deviation

However, the correlation matrix (data are not shown in this article) shows that the highest correlation between two items is 0.66, which does not reflect redundancy. In fact, this alpha score is comparable with one found by de Oliveira Filho and colleagues (Cronbach's Alpha=0.93 for nine items).^18^

### Study limitations and future research

Although we have tried our best to describe and measure a series of psychometric properties of the CCSS as a new scale, there are some limitations that we would like to acknowledge. The CCSS is a self-administered questionnaire, and hence it is prone to social desirability bias. Although the responders are anonymous and their identities remain confidential, trainees may respond to the items in a way socially acceptable to their clinical supervisors or institution. Longitudinal study designs may detect trainees' biases towards clinical supervision.

As this study was a preliminary study for investigating the validity and reliability of the CCSS scale, the validity and reliability evidence of the CCSS may need to be studied more extensively. Further studies using CFA or Rasch analysis are needed to give more robust validity evidence. Besides, other validity evidence such as convergent validity and incremental validity should be developed. A more sophisticated reliability study, such as a multi-faceted Generalisability study, could be used to analyse multiple facets, which are potential causes of error.^36^

We recommend differential person functioning (DPF) using item response theory models (IRT) to identify rogue responses between the observed and expected performance of trainees across 19 items. It is well documented that if a scale does not show a statistically significant degree of DPF, the construct being measured maps onto the scale of interest, providing a reasonable estimation of what we expect to predict about our trainees at the different levels on the subscales of interest.^46^

A further issue is the functioning of response categories of the CCSS. Response categories reflect the construct being measured,^47^ so we recommend inspecting the frequency distribution of the score at the item level to ensure all response categories are plausible using item response curves (IRCs). IRCs would also enable us to detect which parts of groups have the same scores. If a category is rarely used, combining such categories is considered.

Finally, as the scale is newly constructed and utilised a non-random sampling method, which is also used in many studies, further replication studies are required, especially in other countries, to enable generalisation of the results.

**Table 3:** Descriptive statistics of the two CCSS factors (N=388)

Factor	No. Items	Cronbach's Alpha	M (SD)	Skewness	Kurtosis
Factor 1 (SIF)	10	0.93	37.36 (6.02)	-1.14	3.58
Factor 2 (RM)	9	0.89	36.49 (4.39)	-1.65	7.18

M: Mean; SD: Standard deviation; SIF: Supervisory Interaction and Facilitation; RM: Role Modelling

## Conclusions

Using classical test theory and generalisability theory, which is an extension of the classical test theory, our work provides validity and reliability evidence of the CCSS, including its internal structure and internal consistency. However, as this is a new scale, further psychometric studies in different cultures are required to ensure the cross-cultural validity of the CCSS. Other evidence of validity, such as convergent and incremental validity, are also required. IRT analysis (e.g., the Rasch analysis), and CFA are recommended for testing whether the data fit the hypothesised two-factor model of the CCSS.

### Acknowledgements

We thank the Collegium of Cardiology and Vascular Medicine in Indonesia for their support in this study. We would also like to express our appreciation to the Department of Cardiology and Vascular Medicine in eight universities participating in this study and all cardiology trainees for their contribution in the data collection process, as well as the experts contributing in the content validity evaluation of the scale. We also thank Mohammad Iqbal, MD, FIHA of the Department of Cardiology and Vascular Medicine, Faculty of Medicine, Universitas Padjadjaran, Bandung, Indonesia, for his contribution in data collection and study permit in Indonesia. The government of Republic Indonesia supported this work through Indonesia Endowment Fund for Education (LPDP) as the scholarship provider for IJTA.

### Conflict of Interest

The authors declare that they have no conflict of interest.
